# A highly contiguous *de novo* genome assembly of the Mexican Duck (*Anas diazi*) reveals fine-scale structural variation within the mallard complex

**DOI:** 10.1186/s12864-026-13219-3

**Published:** 2026-07-28

**Authors:** Patricia Padilla-Aguilar, María Guadalupe Bravo-Vinaja, Edmundo Gutiérrez-Morales, David Colón-Quezada, Gastón Contreras-Jiménez, Marco Tulio Solano-De la Cruz

**Affiliations:** 1 Postgrado en Ganadería, Colegio de Postgraduados Campus Montecillo, Texcoco de Mora, Estado de México México; 2 Dirección del APFF Ciénegas del Lerma, Comisión Nacional de Áreas Naturales Protegidas, San Felipe Tlalmimilolpan, Toluca de Lerdo, Estado de México México; 3 Instituto de Ecología, Universidad Nacional Autónoma de México, Ciudad Universitaria, Ciudad de México, México

## Abstract

The Mexican Duck (*Anas diazi*) is a quasi-endemic North American dabbling duck whose evolutionary history is marked by recent Pleistocene divergence and ongoing introgressive hybridization with the Mallard (*Anas platyrhynchos*). Because the two species are morphologically and genomically very similar, identifying where their genomes differ structurally is central to understanding how species integrity is maintained under gene flow. Here we present the first highly contiguous de novo genome assembly of *A. diazi* (PacBio HiFi) and use it to map chromosomal inversions across the phylogeny of the mallard complex. k-mer profiling revealed high heterozygosity (1.08%), and read-depth haplotype purging recovered an accurate 1.19 Gb haploid consensus (BUSCO 95.7%). Using independent, orientation-aware approaches—including read-based structural-variant detection and phylogenetic polarization—we find that structural differences between *A. diazi* and the Mallard are numerous but small (kb-scale), and strongly enriched on the Z chromosome. Loci within these species-specific inversions (e.g., PIK3C3, RIT2, and DIPK2B) exhibit high structural protein conservation, while functional annotation highlights post-transcriptional mechanisms. Furthermore, the capture of master non-coding RNAs within these rearrangements, including the spliceosomal U1 and the nucleolar SNORA5, provides a mechanism for species-specific alternative splicing and translational control. Ultimately, we find no evidence for macro-inversion ‘supergenes’; instead, the Mexican Duck maintains its divergence through fine-scale, Z-concentrated and post-transcriptional rewiring, consistent with the genomic architecture of recently diverged, hybridizing lineages.

**Supplementary Information:**

The online version contains supplementary material available at https://doi.org/10.1186/s12864-026-13219-3.

## Introduction

The Anatidae family comprises approximately 145 species of waterfowl worldwide, 33 of which occur in Mexico [1, 2]. Among these, six species are considered resident, including the Mexican Duck (*Anas diazi*) [2]. Many authors consider it the only Mexican endemic duck [3–7]; however, given its distribution, it is a quasi-endemic species, as most of its population lives in Mexico (98%) and a small fraction in the United States (2%) [8]. *A. diazi* is distributed year-round in the inland wetlands of the Mexican Plateau and is currently listed as threatened under Mexican conservation legislation (NOM-059-SEMARNAT-2010) [9]. Population estimates suggest approximately 55,500 individuals worldwide, reflecting its restricted distribution and conservation relevance [10].

The Mexican Duck belongs to the tribe Anatini and is a dabbling duck of shallow freshwater wetlands with omnivorous feeding behavior [11]. Morphologically, *A. diazi* closely resembles the female Mallard (*Anas platyrhynchos*), a similarity that has historically contributed to taxonomic uncertainty [12]. For decades, *A. diazi* was treated as a subspecies of *A. platyrhyncho*s or as part of a North American complex with clinal variation [13]. Phylogenetic [14], morphological, and behavioral evidence accumulated over recent decades led the American Ornithological Society to recognize *A. diazi* as a distinct species in 2020 [15]. Recent studies of dabbling ducks highlight complex evolutionary dynamics—recent Pleistocene divergence, incomplete lineage sorting, and recurrent introgressive hybridization—underscoring the importance of high-resolution genomic data to resolve species boundaries [16–18].

Despite its ecological and evolutionary relevance, *A. diazi* remains one of the least studied Holarctic waterbirds from a genomic standpoint. Long-read technologies such as PacBio HiFi now enable de novo assemblies of unprecedented contiguity for birds [19], whose genomes are generally small, compact, and structurally conserved (0.9–1.3 Gb) with relatively low repetitive content [20–22]. However, assembling wild, outbred avian genomes poses specific challenges: high heterozygosity can cause divergent alleles to be assembled as separate primary contigs, inflating genome size and obscuring true structural variation [23]. Rigorous haplotype purging and k-mer profiling are therefore essential to obtain a reliable haploid consensus suitable for evolutionary analysis [24].

*A. diazi* is embedded in the New World “mallard complex,” a cluster of recently diverged, monochromatic dabbling ducks — including the mottled duck (*A. fulvigula*) and the American black duck (*A. rubripes*) — among which shared ancestry and gene flow are pervasive [13, 16, 18]. Where their ranges meet in the southwestern United States, *A. diazi* and the Mallard (*A. platyrhynchos*) hybridize and backcross, generating recurrent bidirectional introgression, although advanced-generation hybrids remain comparatively uncommon [17, 18]. In such scenarios of recent divergence under ongoing gene flow, chromosomal inversions are of particular interest: by locally suppressing recombination in heterokaryotypic individuals, they can shelter co-adapted alleles from introgression [25]. Detecting and polarizing genuine inversions in fragmented de novo assemblies, however, demands orientation-aware methods to prevent contig-strand artifacts from masquerading as massive rearrangements [26–28].

In birds, the genomic regions that most effectively resist this homogenization are disproportionately located on the Z chromosome. Owing to its reduced effective population size, the hemizygous exposure of recessive incompatibilities in females, and its restricted recombination (the “Fast-Z” effect), the Z diverges faster than the autosomes and concentrates loci that maintain species boundaries under gene flow [29]. This is precisely the pattern reported in the mallard complex, where the Z is severalfold more differentiated between *A. diazi* and the Mallard than the autosomes, despite ongoing hybridization [17]. Because *A. diazi* and the Mallard are otherwise genomically very similar, the informative question is therefore not whether one harbors large rearrangements, but precisely where — and in which lineage — their genomes diverge, and whether that divergence, structural and nucleotide alike, is concentrated on the Z.

Here we present the first highly contiguous *de novo* genome of *A. diazi* and integrate three independent, orientation-aware analyses to chart chromosomal inversions across the phylogeny of the mallard complex: (i) syntenic inversion calling against an outgroup, (ii) read-based structural-variant validation directly from *A. diazi* HiFi reads, and (iii) phylogenetic polarization. Crucially, we couple this structural genomic framework with a functional annotation of these species-specific rearrangements *to elucidate their role* in maintaining species integrity despite ongoing gene flow [25, 30].

## Materials and methods

### Genome size estimation, de novo assembly, and curation

Haploid genome size, heterozygosity, and duplication levels were estimated assembly-free from a k-mer (k = 21) frequency profile using Jellyfish [31] and GenomeScope v2.0 [32], providing a robust genomic ‘ground truth’. De novo assembly was performed using the haplotype-aware assembler hifiasm v0.25.0 [33] to avoid chimeric collapse of highly heterozygous regions. Redundant contigs were subsequently removed using purge_dups v1.2.6 [34], systematically compacting the assembly to its true biological haploid size. Assembly completeness was benchmarked before and after purging using BUSCO v5.5.0 against the aves_odb10 lineage dataset [35]. To characterize the repetitive landscape, a species-specific repeat library was constructed de novo with RepeatModeler v2 [36] and used to mask the assembly via RepeatMasker v4.1.5 [37].

### Reference orientation and orthology

Because de novo contigs are inherently emitted on arbitrary strands, the curated *A. diazi* scaffolds (as well as those of all other analyzed species) were computationally oriented and ordered against the chromosome-level *A. platyrhynchos* reference (ZJU1.0; GCF_015476345.1) [38] using RagTag v2 [39]. RagTag re-orients and orders whole contigs to a reference without altering the internal sequence, effectively eliminating whole-contig flip artifacts while perfectly preserving genuine intra-contig biological inversions. Downstream analyses focused on macrochromosomes 1–4 and the Z chromosome, using the Northern Shoveler (*Spatula clypeata*) as the nearest available outgroup to the genus Anas and the Muscovy Duck (*Cairina moschata*) as a more distant outgroup to reliably root the phylogeny.

### Orientation-aware synteny and inversion detection

Whole-genome alignments were computed per chromosome using minimap2 (asm20, --eqx ) [40]. Inversions were subsequently called using SyRI [27], a tool that rigorously scores rearrangements within their broader syntenic context rather than relying on raw coordinate orientation [41]. This methodological shift replaces the legacy nucmer/MUMmer procedure—where blocks were naively classified as inverted simply if the query start exceeded the query end (S2 > E2)—which we demonstrate conflates arbitrary contig strandedness with biological inversions in fragmented assemblies. For each species, inversions were detected against the Spatula outgroup; thus, the collinear state (0) corresponds to the ancestral/outgroup orientation, and the inverted state (1) represents the derived orientation.

### Read-based validation of Mexican Duck–Mallard inversions

To validate structural variants completely independently of the de novo assembly process, raw *A. diazi* HiFi reads were aligned directly to the Mallard ZJU1.0 reference using minimap2 (map-hifi). Structural variants were then called using Sniffles2 [42]. Inversion calls were rigorously filtered to generate a high-confidence ‘credible-fixed’ set, requiring a PASS status, precise breakpoint resolution, and a variant allele fraction (VAF = supporting reads / total reads) consistent with a fixed homozygous state. This read-based set captures true structural differences between the *A. diazi* reads and the Mallard reference, agnostic to the specific lineage in which they arose.

### Phylogeny and polarization of inversions

A genus-level phylogeny comprising eight anatids was reconstructed from genome-wide Mash sketches [43] utilizing neighbor-joining, explicitly rooted on *Cairina moschata*. To provide an internal control for assembly-contiguity bias in inversion detection, two *A. platyrhynchos* assemblies were included — the chromosome-level ZJU1.0 reference and the more fragmented draft CAU genome. Inversions detected per assembly against the *Spatula* outgroup were clustered into orthologous events based on reciprocal overlap. The ancestral versus derived state of each cluster was subsequently reconstructed via Fitch parsimony [44] across the phylogenetic tree. Consequently, each cluster was assigned a discrete polarization (e.g., derived in a specific terminal lineage, ancestral to a clade, homoplastic, or shared with outgroups). A high-confidence subset was defined by strictly limiting the data to intra-contig inversions ≥ 10 kb, which are intrinsically immune to the aforementioned whole-contig orientation artifacts.

Enrichment of *A. diazi*-derived inversions on the Z chromosome was assessed using a one-sided binomial test, with the null probability set to the fraction of the analyzed macrochromosome sequence (chromosomes 1–4 and Z; ZJU1.0 lengths) contributed by the Z.

### Coordinate liftover and gene annotation

Because the polarization coordinates were inherently expressed in the Spatula reference frame and the curated *A. diazi* inversions in native *A. diazi*-contig coordinates, all genomic intervals were lifted over to the common Mallard frame. This was achieved by realigning each inversion sequence to ZJU1.0 [38] with minimap2 and extracting the best-supported target interval (utilizing a mapping fraction, map_frac, as a quantitative confidence metric; intervals with map_frac < 0.20 were discarded as unreliable). Genes physically overlapping each lifted interval were annotated using the ZJU1.0 RefSeq GFF [45]. This robust, sequence-based liftover procedure is frame-independent and ensures the three parallel analyses are directly comparable regarding gene content.

### Three-way comparison

Three distinct inversion sets were compared based on shared gene content within the common Mallard frame: (A) the read-based Mexican Duck–Mallard set (derived from Sniffles2); (B) the manually curated set of 64 *A. diazi* inversions (assembly-based); and (C) the genus-wide polarized set. Overlaps were assessed strictly at the level of annotated gene symbols (using custom Python scripts and bedtools intersect [46]), and each shared gene was flagged according to whether its phylogenetic polarization explicitly implicated the *A. diazi* evolutionary lineage.

### Functional annotation of coding sequences and protein conservation

To assess whether structural variants disrupt open reading frames or drive protein innovation, we extracted the nucleotide sequences of all high-confidence inversions and evaluated their coding capacity. Encapsulated genes were queried against the UniProt [47] and Mallard proteome databases using sequence-alignment heuristics [48]. For each trapped coding sequence, structural protein conservation was quantified as the precise percentage of sequence identity (pident) against its *A. platyrhynchos* ortholog. Genes localized to the Z chromosome were specifically flagged to evaluate the differential impact of structural variation on sex-linked loci, given their known role in avian speciation [49].

### Identification of non-coding RNAs within *A. diazi* inversions

To identify structural RNAs captured by these rearrangements, exact inversion sequences were scanned against the Rfam database (v14.0) [50] using the covariance model-based search tool Infernal (cmscan v1.1.5) [51]. High-confidence hits (E-value < 1e-10) were retained to document the specific capture of master regulatory ncRNAs, notably the spliceosomal U1 and nucleolar SNORA5.

### Integrative visualization and dual-genome synteny mapping

Data integration and visualizations were performed using custom Python scripts that employed the pandas [52], matplotlib [53], seaborn [54], and pycirclize libraries [55]. All custom R and Python scripts utilized for structural variant density and visualization are provided in Additional File 5. To synthesize the evolutionary impact of inversions and demonstrate macro-collinearity, a high-resolution dual-genome Circos plot was constructed. This synteny map projected the physical alignments between the *A. diazi* curated assembly and the Mallard reference (ZJU1.0), visually distinguishing perfect collinear alignments from inverted blocks. Crucially, the outer tracks of the Circos plot were populated with the functional landscape data, mapping the precise physical locations of the *A. diazi*-specific inversions and the structurally captured master ncRNAs directly onto the macrochromosomes. Additionally, two functional profiles were generated: (1) a global landscape comparing inversion span against transposable element density; (2) a coding conservation scatter plot displaying protein identity versus inversion size.

## Results

### Genome size, heterozygosity, and assembly curation

**Fig. 1:**
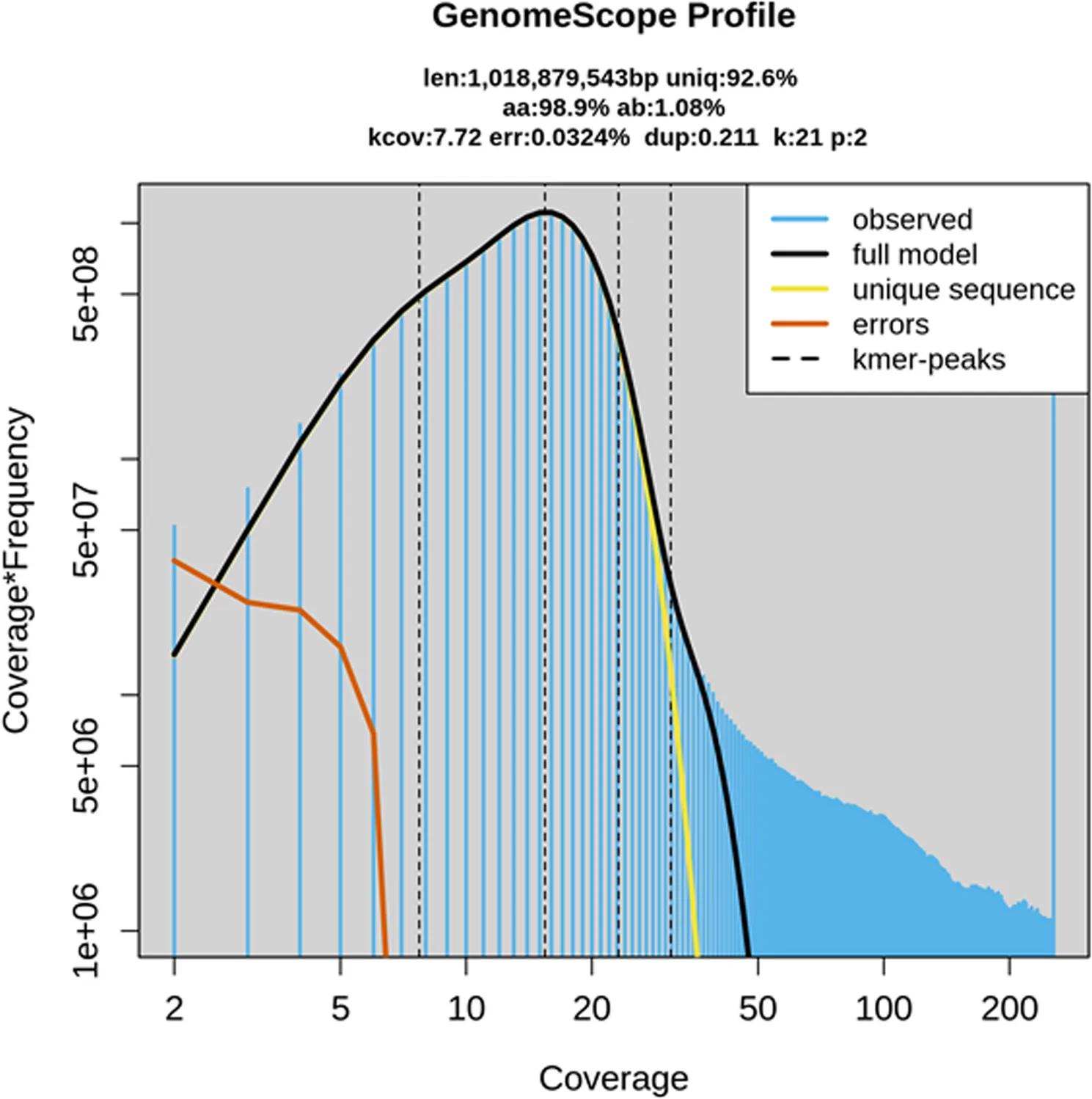
K-mer frequency distribution and genome size estimation of *Anas diazi*. The GenomeScope profile illustrates the 21-mer depth distribution derived from raw PacBio HiFi reads. The topological peaks reflect the genomic complexity and high heterozygosity of the wild Mexican Duck, providing high-confidence baseline estimates of total genome length, repetitive content, and optimal read-depth thresholds used during subsequent de novo assembly and haplotype-purging processes

GenomeScope modeling of k-mer frequencies estimated a haploid genome size of 1,017.7–1,020.1 Mb with moderate heterozygosity (1.07–1.09%) and ~ 75.5 Mb of repetitive sequence, exhibiting an excellent model fit (96.3–99.7%; Fig. 1). The initial primary assembly spanned 1,593.94 Mb across 4,540 scaffolds (N50 = 876 kb). Although it was 96.4% BUSCO-complete, it showed 28.4% duplicated orthologs—a diagnostic hallmark of allelic splitting driven by high heterozygosity in the sequenced individual. Following curation with purge_dups, the assembly collapsed to a highly contiguous 1,195.99 Mb (1.19 Gb) across 2,432 scaffolds, with an improved N50 of 1.0 Mb (Table 1). This curated version retained 95.7% complete BUSCOs, while the duplicated fraction fell drastically to 0.8% (Additional File 4). This targeted reduction successfully removed technical redundancy without eroding the functional gene space, yielding a high-fidelity haploid reference for *A. diazi*.

**Table 1:** Assembly metrics for the *Anas diazi* genome before and after read-depth haplotype purging

Metric	Primary (uncurated)	Curated (post purge_dups)
Total length	**1,593.94 Mb**	**1,195.99 Mb (1.19 Gb)**
Scaffolds	**4,540**	**2,432**
Scaffold N50	**876 kb**	**1.0 Mb**
BUSCO complete (C)	**96.40%**	**95.70%**
BUSCO duplicated (D)	**28.40%**	**0.80%**
BUSCO single-copy (S)	**68.00%**	**94.90%**
Heterozygosity (k-mer)	**1.08%**	**—**

### Repetitive landscape in evolutionary stasis

Repetitive elements occupy approximately 7.3–7.4% of the *A. diazi* genome (~ 75.5 Mb). This fraction is essentially identical to that of the Mallard and is consistent with the characteristically lean architecture of avian genomes [56]. This parity provides crucial evidence that the recent Mexican Duck–Mallard evolutionary split was not accompanied by transposable-element bursts. Consequently, comparative structural signals downstream are highly robust and unlikely to be confounded by lineage-specific repeat expansions.

### Orientation-aware synteny resolves fragmented assembly artifacts

**Fig. 2:**
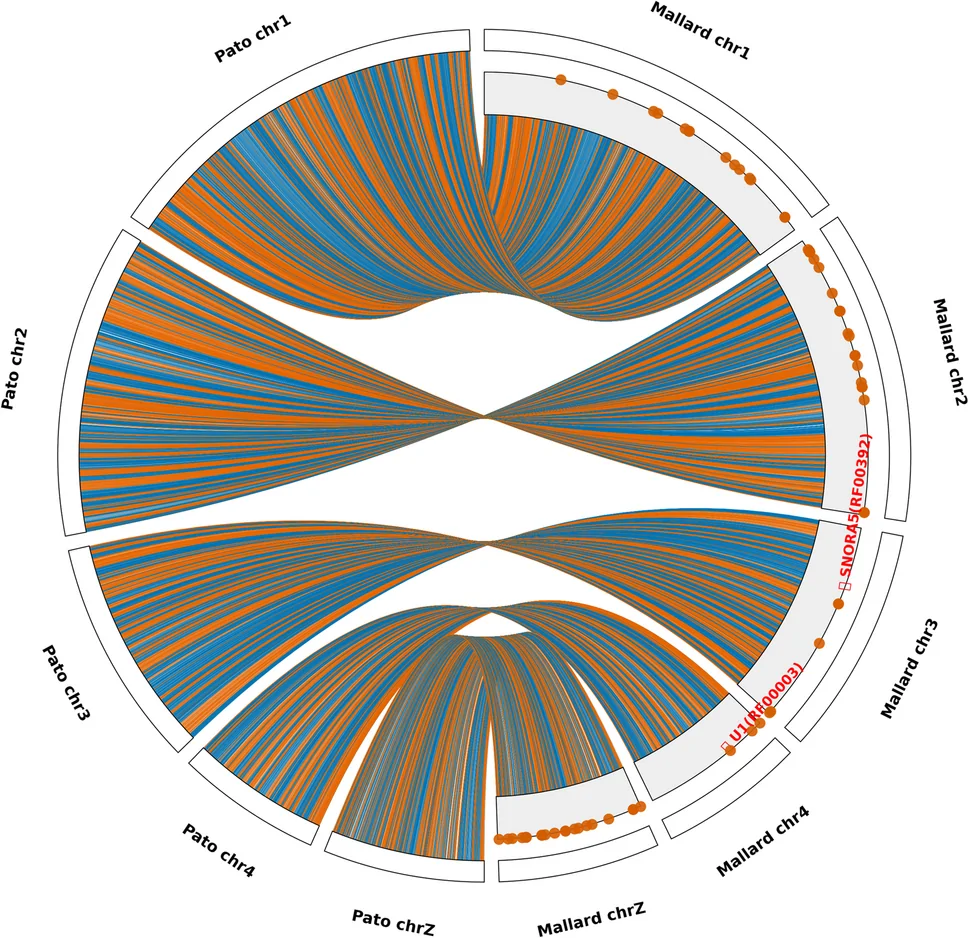
Dual-genome macro-collinearity between the Mexican Duck (*Anas diazi*) and the Mallard (*Anas platyrhynchos*). High-resolution circos plot demonstrating overwhelming chromosomal synteny across the two lineages. Chromosomes are laid out circumferentially; inner ribbons depict genomic alignments and structural orthology. The outermost functional track highlights the genomic distribution of targeted non-coding RNA (ncRNA) loci caught within micro-rearrangements

Initial coordinate-orientation analysis of whole-genome alignments suggested massive inverted blocks—comprising ~ 49–51% of the aligned sequence—particularly on macrochromosomes 1–3. However, because de novo contigs are emitted on arbitrary strands in highly fragmented assemblies, traditional coordinate-based metrics often conflate technical contig flips with true biological inversions. When contigs were computationally oriented to the reference using RagTag, and inversions were rigorously scored within their syntenic context using SyRI, the massive rearrangement signal completely disappeared. As visualized in our high-resolution dual-genome synteny plot (Fig. 2), *A. diazi* is overwhelmingly macro-collinear with the Mallard. Uninterrupted syntenic blocks dominate the genomic landscape, demonstrating that structural variation between these species is strictly restricted to fine-scale, localized inversions rather than massive chromosomal restructuring.

### Read-based Mexican Duck–Mallard inversions are small and Z-biased

To validate these fine-scale structural variants, Sniffles2 calling of *A. diazi* HiFi reads against the ZJU1.0 reference yielded a high-confidence set of 20 fixed inversions (median Variant Allele Frequency = 0.70). Additional File 2: column definitions provided in Additional File 3. These events are strictly kb-scale (median length 18.3 kb; range 1.2 kb to a few hundred kb, with the largest values likely reflecting microchromosome or scaffold-edge technical effects). They are unevenly distributed across chromosomes 1 (5 events), Z (4), 3 (3), and 2 (2), as well as several microchromosomes. Notably, no megabase-scale fixed inversion distinguishing the two species was recovered, perfectly corroborating the macro-syntenic reassessment presented in 3.3.

### Phylogenetic polarization: an asymmetric structural history

Mash neighbor-joining recovered the expected topology for the eight analyzed anatids: *Cairina* (root), followed by *Spatula*, *A. crecca*, *A. acuta*, and finally a mallard-complex clade comprising *A. diazi*, *A. zonorhyncha*, and *A. platyrhynchos*. This topology is congruent with previously published multilocus and genomic phylogenies of the mallard complex [13, 57, 58], validating the distance-based framework used here for polarization.

Across the genus, Fitch parsimony polarized 1,300 inversion clusters, which resolved into distinct evolutionary categories: 1,027 (79%) were derived on a single terminal lineage, ~ 105 mapped to internal ancestral nodes shared by two or more *Anas* species, 116 (9%) were homoplastic—independently re-derived in unrelated lineages—and 52 were also present in the outgroups and thus non-polarizable. The recurrence of homoplastic inversions in the same genomic regions across species indicates that particular loci are structurally labile and repeatedly targeted by inversion, rather than each event being unique.

Among terminal lineages, the raw, unfiltered counts were strongly asymmetric (Table 2): 885 on *A. platyrhynchos*, 52 on *A. acuta*, 41 on *A. crecca*, 27 on *A. diazi*, and 11 on *A. zonorhyncha*. This asymmetry is only partly biological; it is substantially inflated by reference-contiguity bias because the chromosome-level mallard assembly can express and resolve large inversions that fragmented assemblies from the other species cannot capture. The clearest evidence is internal to *A. platyrhynchos* itself: the chromosome-level ZJU1.0 yields 827 raw derived inversions, whereas the draft CAU assembly of the same species yields only 58.

**Table 2:** Phylogenetic polarization and lineage-specific derived inversions across the mallard complex

Lineage	Raw derived inversions	High-confidence derived inversions	Z High-confidence derived inversions	Notes
*A. platyrhynchos* (ZJU1.0)	**827**	**20**	**5**	**Chromosome-level ref (contiguity-inflated raw)**
*A. platyrhynchos* (CAU)	**58**	**35**	**10**	**Draft assembly (contiguity control)**
*A. acuta*	**52**	**12**	**3**	**Fragmented assembly**
*A. crecca*	**41**	**14**	**3**	**Fragmented assembly**
*A. diazi* (Mexican Duck)	**27**	**10**	**5**	**13 on raw chromosome Z**
*A. zonorhyncha*	**11**	**2**	**2**	**Fragmented assembly**

**Fig. 3:**
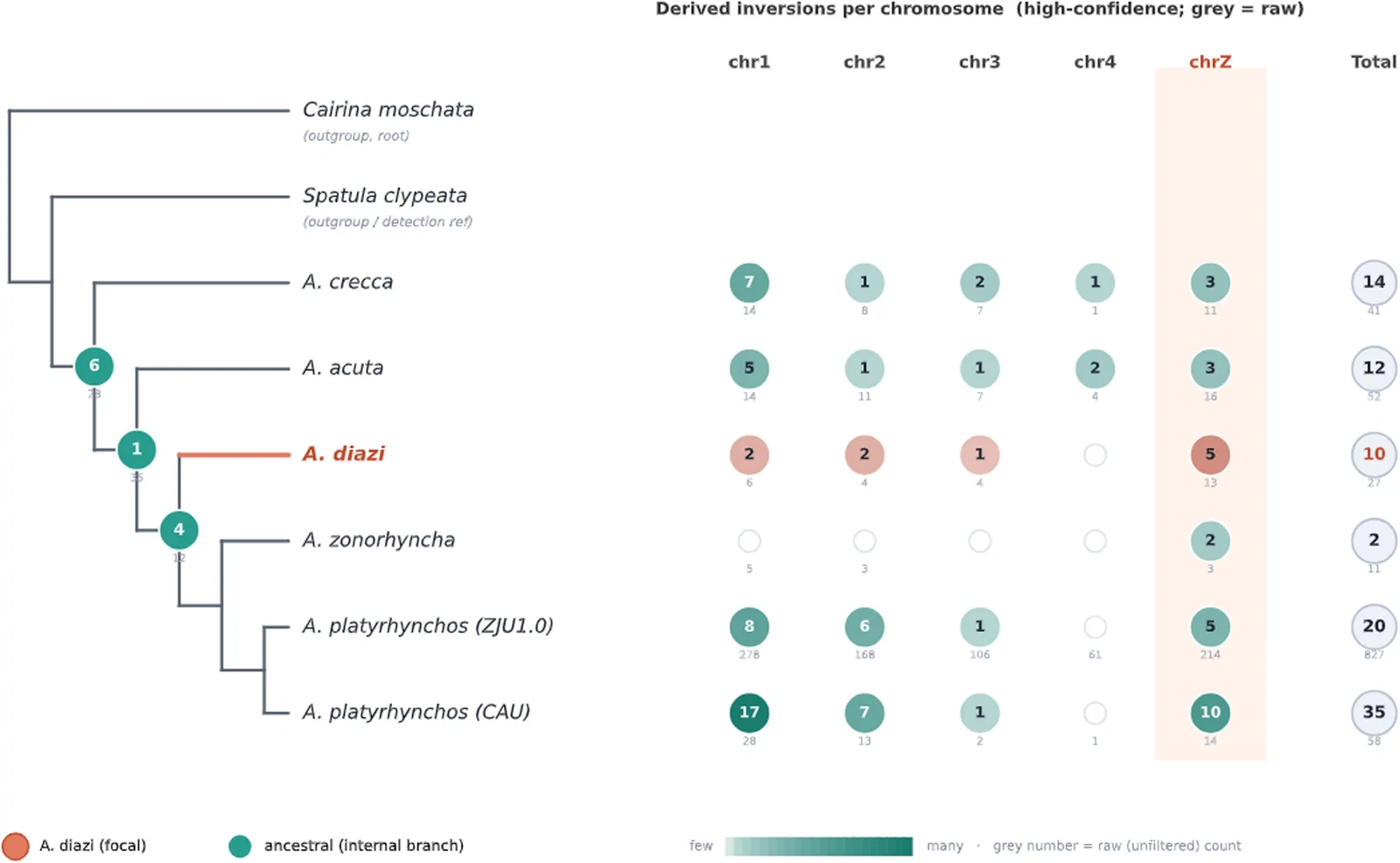
Chromosomal inversions polarized across the phylogeny of the mallard complex. Neighbor-joining tree from genome-wide Mash distances (eight anatids), rooted on *Cairina moschata*; inversions were detected per assembly against the *Spatula clypeata* outgroup (SyRI) and their ancestral/derived state reconstructed by Fitch parsimony. The right panel shows, for each terminal lineage and chromosome (chr1–4 and Z), the number of high-confidence derived inversions (intra-contig, ≥ 10 kb; circle shading scaled to count), with the raw unfiltered count in grey below each circle; the Total column gives the per-lineage sum. Teal circles on internal branches give the number of inversions arising on each ancestral branch. *Anas diazi* (red) carries 10 high-confidence derived inversions, 5 of them on chromosome Z. Two *A. platyrhynchos* assemblies are shown — the chromosome-level ZJU1.0 and the draft CAU genome — whose large difference in raw counts (827 vs. 58) collapses to comparable high-confidence counts (20 vs. 35), illustrating that the apparent between-lineage asymmetry is driven largely by assembly contiguity rather than biology

Under the high-confidence filter (intra-contig, ≥ 10 kb), these counts collapse to small, comparable values—55 in *A. platyrhynchos* (ZJU1.0 20 + CAU 35), 14 in *A. crecca*, 12 in *A. acuta*, 10 in *A. diazi*, and 2 in *A. zonorhyncha*—and the raw asymmetry largely disappears (Fig. 3). The structural differences between Mexican Duck and the Mallard are therefore few and small, and the apparent excess on the mallard branch is principally a contiguity artifact rather than genuine biological acceleration.

Partitioning the high-confidence inversions by chromosome (Fig. 3) shows a markedly non-uniform distribution: events concentrate on chromosome 1 and the Z, are sparse on chromosomes 3 and 4, and—relative to its physical size—the Z is disproportionately affected in every lineage. *A. diazi* stands out as the only lineage with appreciable counts in which the Z carries more derived inversions than any autosome (5 on the Z versus ≤ 2 per autosome; 50% of its high-confidence events), a stronger Z-bias than in *A. acuta*, *A. crecca* or either *A. platyrhynchos* assembly (21–29% on the Z). This pattern is consistent with the established role of the avian Z chromosome as a hotspot of lineage differentiation in the mallard complex.

### *A. diaz*i-derived inversions are few, Z-enriched, and gene-poor

Because our polarization detects inversions against the *Spatula* outgroup rather than against the Mallard, an *A. diazi*–“derived” inversion here denotes an event that arose on the *A. diazi* lineage relative to the inferred ancestral (outgroup) state — a genus-wide evolutionary polarization, and thus conceptually distinct from the pairwise *A. diazi*-versus-Mallard differences catalogued directly from the HiFi reads (§ 3.4). Under this framework, 27 inversions are uniquely derived in *A. diazi* in the raw set, 13 of which (48%) map to the Z chromosome; the high-confidence, intra-contig subset (≥ 10 kb) comprises 10 events, 5 of them Z-linked. This Z-bias is statistically significant: although the Z represents only 12.9% of the analysed macrochromosome sequence (chromosomes 1–4 and Z), it harbours 50% of the high-confidence *A. diazi*-derived inversions — a 6.7-fold higher density than the autosomal average (0.0592 vs. 0.0088 inversions Mb⁻¹; one-sided binomial test, *P* = 0.0052; Fig. 3), consistent with the Z acting as a disproportionate hotspot of lineage differentiation in the mallard complex [49]. Reassuringly, the independent read-based set (Sniffles2, called directly against the Mallard reference; 3.4) is concordant, with the Z among the most affected chromosomes — a convergence across two different reference frames that reinforces the signal.

These specific inversions are compact and overwhelmingly gene-poor; almost all exclusively overlap uncharacterized loci (LOC models). The sparse few that encompass named genes represent compelling evolutionary candidates—such as chrZ_inv962, spanning PIK3C3 and RIT2, and a chromosome 2 event spanning NUP153. Crucially, however, no *A. diazi*-derived inversion encapsulates a massive block of co-adapted, named genes that would typify a classical ‘supergene’ architecture [59].

### The curated assembly set and a corroborated core

**Fig. 4:**
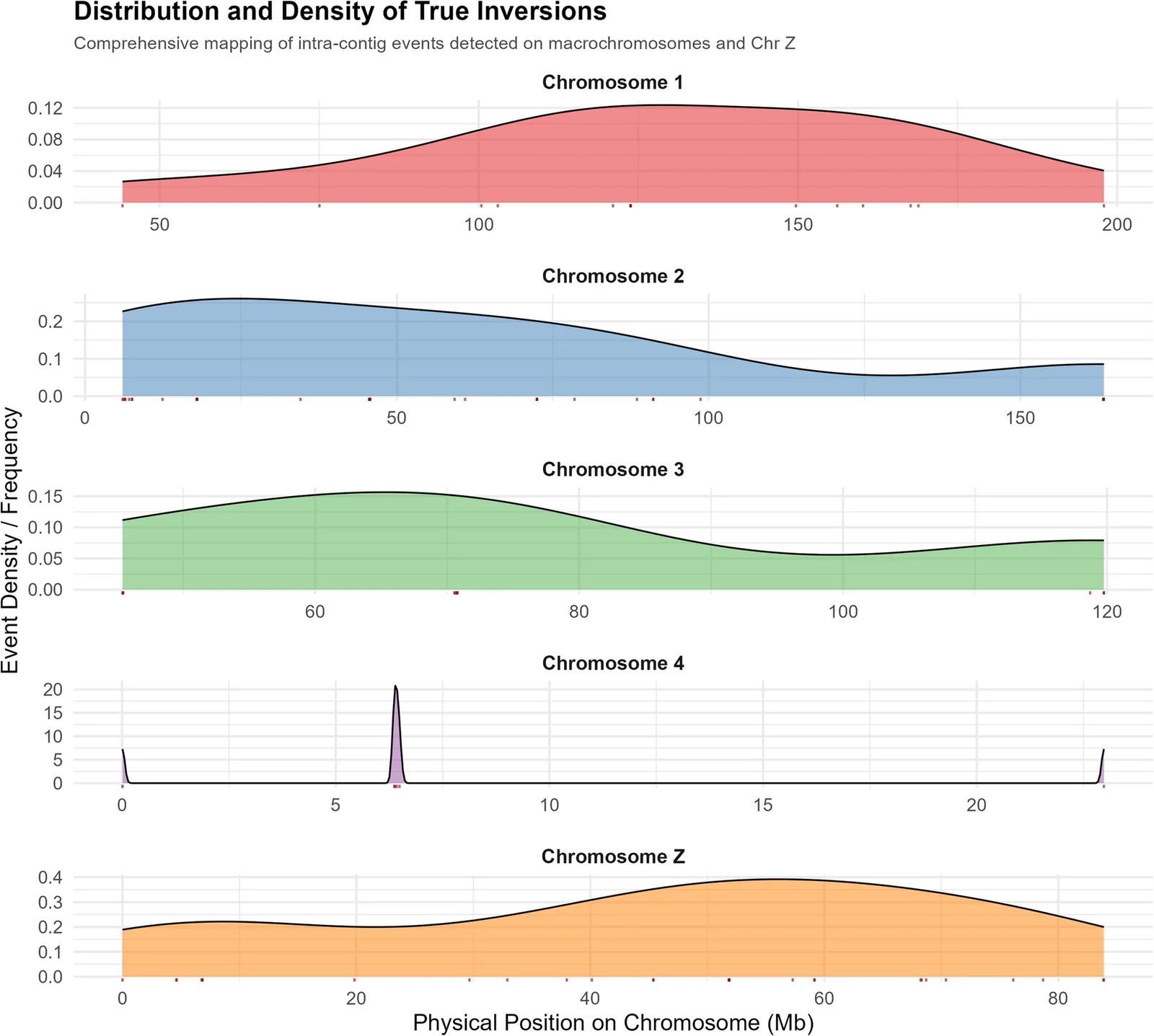
Heterogeneous genomic distribution and density of the 64 validated chromosomal inversions. Kernel density estimation profiles across the macrochromosomes of *Anas diazi*. The curves illustrate a non-uniform accumulation of true intra-contig structural variants, showing pronounced spatial clustering and enrichment within chromosome 2 and the Z chromosome rather than a random uniform distribution across the genome

A manually curated set of 64 *A. diazi* inversions (ranging from 10 to 461 kb, median 20 kb) further mirrors the Z-bias, showing marked enrichment on chromosomes 2 (22 inversions) and Z (20) (Fig. 4; Additional File 1). Within this set, 30 inversions contain at least one gene (25 named), including functional candidates such as SETD2, REV3L, PLK2, GCNT1, RORB, CCR6, and NBEAL2 within the larger rearranged blocks. When triangulating the three independent analyses based on shared genes in the Mallard reference frame, the curated set overlaps with the read-based Mexican Duck–Mallard set at the DIPK2B locus and an additional Z-linked locus. Furthermore, it overlaps with the genus-wide polarized set at 46 genes (14 named). Importantly, the genes shared between the polarization and the read-based set fall within inversions polarized to the Mallard (or to ancestral/homoplastic states) rather than to *A. diazi.* Consequently, only a highly restricted core—best exemplified by DIPK2B and the Z-linked locus—is robustly corroborated by two fully independent methodologies (assembly-based and read-based mapping), constituting the most strictly defensible set of true Mexican Duck–Mallard structural differences (Table 3).

**Table 3:** High-confidence candidate genes located within corroborated *Anas diazi*-derived inversions

Analysis	Scope/method	Key outcome
Read-based (Sniffles2)	***A. diazi*** ** HiFi reads vs. Mallard ZJU1.0**	**20 fixed inversions, kb-scale, Z-biased**
Curated assembly set	**64 manually curated ** ***A. diazi*** ** inversions**	**10–461 kb; enriched chr2 & Z; 25 named genes**
Phylogenetic polarization	**8 anatids vs. Spatula; Fitch**	**27** ***A. diazi*-derived (13 on Z); gene-poor**
Corroborated core	**curated ** *∩* **read-based**	**DIPK2B + a Z-linked locus (independent support)**
Candidate genes	A. ***diazi*-derived / curated**	**PIK3C3**,** RIT2**,** NUP153**,** SETD2**,** REV3L**,** PLK2**

### High protein conservation within species-specific inversions

Given the strictly fine-scale nature of these structural differences, we investigated the coding capacity of the high-confidence *A. diazi*-specific inversions. Despite acting as localized recombination modifiers, our data show that these structural variants are not driving divergence through rapid protein-coding mutations. Sequence analysis of the loci physically trapped within these rearranged regions (including the highly corroborated Z-linked candidates PIK3C3 and RIT2, as well as the DIPK2B core) revealed extreme structural stasis. These proteins exhibit > 95% amino acid sequence identity when compared to their *A. platyrhynchos* orthologs. This striking conservation indicates that the speciation process maintaining the Mexican Duck distinct from the Mallard is largely uncoupled from de novo protein innovation.

### Non-coding RNAs captured within *A. diazi* inversions

**Fig. 5:**
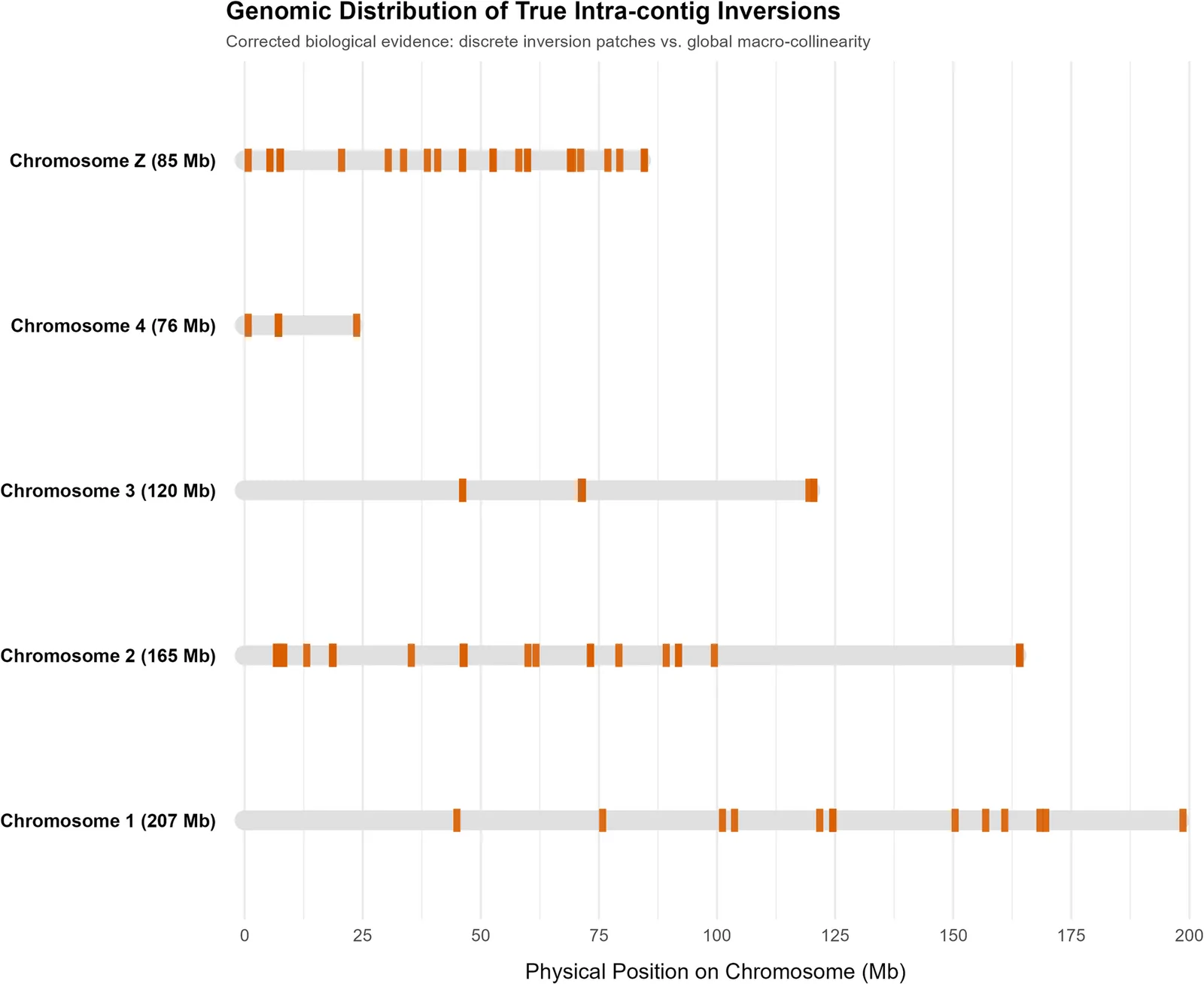
Physical topographic map of corroborated chromosomal inversions on chromosomes. Ideogram representing the precise physical coordinates of verified structural mutations. Arrows indicate the precise genomic capture and encapsulation of master non-coding RNA regulators, highlighting the spliceosomal U1 RNA within a rearranged block on chromosome 3 and the small nucleolar RNA SNORA5 trapped on chromosome 2

Crucially, scanning the precise boundaries of these inversion sequences against the Rfam database revealed the targeted capture of master non-coding RNAs (ncRNAs) within the rearranged blocks (Fig. 2, outer ring). Highly significant hits (E-value < 1e-10) confirmed that *A. diazi*-specific inversions encapsulate critical structural regulatory RNAs, most notably the spliceosomal U1 (chromosome 3) and the nucleolar SNORA5 (chromosome 2) (Fig. 5). The encapsulation of these ncRNAs within *A. diazi*-specific inversions suggests that divergence between the two species may involve post-transcriptional regulation rather than changes to protein-coding sequence alone. This interpretation is consistent with the protein-sequence conservation reported in 3.8, though confirming a regulatory effect would require expression-level data.

## Discussion

### Reassessing macro-inversions in fragmented wildlife genomes

Our primary methodological finding highlights a critical pitfall in non-model structural genomics: an initial signal of chromosomal inversions —encompassing nearly half of macrochromosomes 1–3—was entirely an artifact of unoriented contigs rather than genuine evolutionary biology. In any de novo draft assembly, contigs are inherently emitted on arbitrary strands. If comparative pipelines attempt to score synteny directly from raw coordinate orientations (e.g., the classic S_2 > E_2 rule in legacy MUMmer algorithms), roughly 50% of all unoriented contigs will be falsely classified as inverted blocks. Consequently, the calculated “breakpoint density” of the alignment merely reflects the assembly’s underlying contig fragmentation. By implementing an orientation-aware framework—utilizing RagTag scaffolding to resolve strand orientation, followed by SyRI to parse rearrangements strictly within their broader syntenic context—this massive rearrangement signal vanished completely. The macro-collinearity between *A. diazi* and the Mallard was independently corroborated by raw, read-level alignments using Sniffles2, as (Fig. 2). This carries a vital, generalized caution for the wildlife genomics community: claims of macro-inversions or inversion-locked “supergenes” derived from raw, contig-level alignments of fragmented assemblies must be treated with skepticism [19]. They demand rigorous orientation-aware workflows and long-read confirmation before any biological or ecological interpretations are pursued.

### Where two look-alike species actually differ: the Z-chromosome

Because *A. diazi* and the Mallard exhibit exceptional morphological similarity and share a history of ongoing hybridization, the most evolutionarily informative signal lies in the precise catalog of their genomic divergence. Our curated data show that this divergence is characterized not by massive macro-rearrangements but by numerous fine-scale (kb-scale) structural variants, heavily concentrated on the Z chromosome. The sex chromosomes are widely recognized as primary engines of avian speciation, frequently exhibiting accelerated rates of morphological and behavioral differentiation relative to autosomes—a phenomenon driven by a lower effective population size (N_e), restricted recombination in females, and exposed hemizygous selection (the “Fast-Z” effect) [29, 49]. This is particularly evident within the mallard complex, where the Z chromosome consistently exhibits disproportionately higher genetic differentiation (F_ST) than autosomes despite high gene flow [17]. The profound Z-enrichment of *A. diazi*-derived inversions identified here aligns perfectly with this biological paradigm. It establishes that even in the absence of massive megabase-scale chromosomal shielding, the sex chromosomes remain the principal structural theatre where lineage-specific differentiation is initiated and maintained.

### Asymmetry and the structural constraints of single-reference genomics

Phylogenetic polarization is the analytical pivot that provides directionality to the other two analyses: whereas the read-based (Sniffles2) and curated assembly catalogs enumerate *where* the *A. diazi* and Mallard genomes differ, only the genus-wide Fitch reconstruction establishes *which lineage* each difference arose in. Read together, the three analyses converge on a single picture—overwhelming macro-collinearity overlaid by small, Z-biased differences—and intersect on a minimal corroborated core (DIPK2B and a Z-linked locus) supported independently by assembly- and read-based evidence.

At face value, Fitch parsimony assigned far more derived inversions to the *A. platyrhynchos* lineage than to *A. diazi* (Table 2). Still, this asymmetry is largely an artifact of reference contiguity rather than biological acceleration. Because the chromosome-level mallard assembly can resolve large structural variants that the fragmented assemblies of the other species cannot span [26, 42], detection is biased toward the reference branch. The two *A. platyrhynchos* assemblies make this explicit: the chromosome-level ZJU1.0 yields 827 raw derived inversions, whereas the draft CAU genome of the same species yields only 58, and under the high-confidence filter (intra-contig, ≥ 10 kb), the inter-lineage asymmetry collapses to small, comparable values (*A. platyrhynchos* 55, *A. diazi* 10; Fig. 3). Rather than diminishing our findings, this clarifies them: standard Mallard-referenced comparisons are structurally blind to part of the rearrangement landscape of fragmented lineages, so the conservative, intra-contig, Z-biased set of *A. diazi* inversions identified here is the most resilient and genuine structural signature of the Mexican Duck available to date. Resolving the true magnitude of this history will ultimately require chromosome-level, error-corrected assemblies of adjacent *Anas* species [19].

Biologically, the chromosomal distribution of these inversions is itself informative (Fig. 3): beyond a size-dependent autosomal baseline—counts are greatest on the large chromosome 1 and sparse on chromosomes 3 and 4—the Z chromosome carries more inversions than expected from its size, most strikingly in *A. diazi*, where it exceeds every autosome, and several homoplastic inversions also map there. This independent, distribution-based line of evidence reinforces the Z-centered divergence developed in 4.2.

### Evolutionary candidates and the regulatory paradigm

Most notably, this fine-scale structural shuffling has resulted in the targeted encapsulation of master non-coding RNAs (ncRNAs) within the rearranged blocks, prominently displayed in the outer functional ring of our dual-genome visual map (Fig. 2). The precise physical capture of the spliceosomal U1 RNA on chromosome 3 and the small nucleolar SNORA5 on chromosome 2 (Fig. 5) suggests that post-transcriptional splicing fidelity and ribosomal RNA maturation pathways are being structurally sequestered. Rather than relying on de novo protein innovation, the phenotypic and ecological integrity of *A. diazi* in the face of ongoing gene flow appears to be mediated by fine-scale structural variations. By capturing key non-coding elements, these rearrangements likely drive divergence primarily through post-transcriptional control [60]. Among the few named coding loci trapped within these regulatory-dense networks, several high-confidence candidates warrant immediate functional follow-up: the Z-linked PIK3C3 (involved in autophagy and vesicular trafficking) and RIT2 (a crucial neuron-specific GTPase linked to signaling and behavior), alongside the autosomal core component DIPK2B (Table 3). These genes represent highly defensible structural hypotheses for future functional validations.

### Limitations

We explicitly acknowledge that our evolutionary inferences are bounded by the use of a single, albeit highly contiguous, *A. diazi* individual compared against a multi-species dataset of heterogeneous assembly contiguity. Phylogenetic polarization remains inherently conservative for fragmented assemblies and structurally biased toward the reference frame. Furthermore, while our sequence-based liftover pipeline operates independently of coordinate systems, its alignment accuracy inevitably degrades within highly repetitive or low-complexity regions (quantified by lower map_frac metrics); hence, *A. diazi*-derived variants mapping near scaffold boundaries or transposable-element clusters must be interpreted with caution. Finally, because structural polymorphism varies across space and time, the exact fixation rates of these fine-scale inversions and their precise impacts on localized recombination suppression cannot be definitively established from a single reference individual. Population-level low-coverage resequencing across the Anas contact zone represents the imperative next step to validate the macro-evolutionary trajectories of these structural candidates [61].

## Conclusions

We have presented the first highly contiguous, curated de novo genome assembly of the Mexican Duck (*A. diazi*) and used it to construct a comprehensive structural variant map across the phylogeny of the mallard complex. While initial naive coordinate alignments falsely suggested massive, megabase-scale macro-inversions on chromosomes 1–3, our orientation-aware syntenic framework, combined with independent read-based validations, effectively dismantled this technical artifact. Instead, *A. diazi* and the Mallard exhibit an overwhelming macro-collinearity, with their structural divergence restricted to a discrete layer of small, kb-scale inversions that are deeply enriched on the Z chromosome and largely polarized to the Mallard branch. Ultimately, for two exceptionally young and morphologically cryptic sister species, this fine-scale, non-coding, and sex-chromosome-concentrated structural architecture constitutes the genuine genomic signature that delineates them. The validated inversion core, candidate loci, and methodological template established herein provide a robust and precise foundation for future conservation genetics and speciation genomics within the genus Anas.

## Supplementary Information

Additional file 1: Table S1 (curated_investments_summary.csv).

Additional file 2: Data Dictionary (INVESTMENTS _diazi_vs_mallard_DICTIONARY.txt)

Additional file 3: Dataset S1 (INVESTMENTS_diazi_vs_mallard_credible_fixed.csv).

Additional file 4: BUSCO Quality Report 1 Before Purge_Dups.

Additional file 5: BUSCO Quality Report 2 After Purge_Dups.

Additional file 6: Repeat DNA and TEs in A. diazi and A. platyrhynchos.

Additional file 7: Dataset S2 — Genus-wide inversion polarization. The eight anatid genome assemblies analysed and their public accessions. Inversions and Sniffles Data set.

## Data Availability

The data that support the findings of this study are available from the corresponding author upon reasonable request. The Raw data for this study can be found in the NCBI SRA repository with accession number BioProject **PRJNA1212198** , BioSample accession SAMN46294654.
